# Impact of mechanical support with the impella for ventricular septal rupture

**DOI:** 10.1007/s11748-026-02265-z

**Published:** 2026-02-25

**Authors:** Shintaro Okuda, Noriyuki Takashima, Shintaro Okuda, Fumihiro Miyashita, Kohei Hachiro, Kentaro Matsuoka, Taiki Kakiuchi, Chihiro Yokoyama, Suzuki Tomoaki

**Affiliations:** Department of Cardiovascular Surgery, Shiga University of Medical Science, Setatsukinowa-cho, Otsu, Shiga 520-2192 Japan

**Keywords:** Ventricular septal rupture, Extended sandwich patch technique, Acute myocardial infarction, Impella

## Abstract

**Objectives:**

Ventricular septal rupture is a rare but fatal complication of acute myocardial infarction. Although mechanical circulatory support is increasingly being used to stabilize the hemodynamics in such cases, its definitive clinical benefit remains unclear. Delayed surgical intervention after achieving hemodynamic stabilization with mechanical circulatory support is considered to offer better outcomes than emergency surgery. This study aimed to evaluate the efficacy of using the Impella device (Abiomed, Danvers, MA, USA) as a bridge to surgical repair in patients with post-infarction ventricular septal rupture.

**Methods:**

We retrospectively analyzed 12 patients who underwent surgical ventricular septal rupture closure under perioperative Impella support between October 2022 and June 2025 at our institution.

**Results:**

The 30-day mortality was 8% (1/12 patients). The mean duration from admission to surgery was 64 ± 35 h (range 2–118 h). Preoperatively, inhaled nitric oxide (NO) was administered to nine patients (75%). The mean serum lactate concentration was 24.9 mg/dL at presentation and improved to 10.2 mg/dL at the time of surgery (*p*=0.001). One device-related complication was observed. A residual shunt was present in four patients (33%), one of whom required reoperation.

**Conclusions:**

Our findings demonstrated favorable early outcomes with a 30-day mortality of 8%. Impella support facilitated preoperative optimization of the systemic condition and enabled a safe transition to definitive surgical repair. Further investigations are warranted to refine the optimal timing of surgery and to assess long-term outcomes in this challenging population of patients with ventricular septal rupture.

## Introduction

Ventricular septal rupture (VSR) following acute myocardial infarction (AMI) remains a catastrophic mechanical complication with a poor prognosis. Despite advances in surgical techniques, the perioperative mortality rates of VSR are high [1]. Optimal preoperative management balances the risk of suture dehiscence associated with early intervention against the potential for clinical deterioration during delayed VSR repair.

The introduction of the Impella percutaneous left ventricular assist device (Abiomed, Danvers, MA, USA) has enabled more effective ventricular unloading. Increasing interest has focused on the role of the Impella as a bridge-to-surgery strategy for VSR [2]. In contrast to an intra-aortic balloon pump (IABP) or venoarterial extracorporeal membrane oxygenation (ECMO), the Impella directly ejects blood from the left ventricle, thereby reducing the afterload and myocardial oxygen consumption. However, clinical evidence regarding the application of the Impella in patients with VSR remains scarce, and there is no consensus on the optimal timing of Impella implantation, perioperative management, and impact on surgical outcomes.

In the present study, we conducted a retrospective analysis of patients with VSR treated at our institution under Impella support. We further evaluated the clinical significance of Impella implantation and discuss the potential implications for future therapeutic strategies.

## Methods

At Shiga University of Medical Science Hospital, formal implementation of the Impella device commenced in January 2021. Since then, the Impella has been used to optimize the preoperative hemodynamics in patients with VSR. The standard surgical approach for VSR at our institution is the extended sandwich patch technique, with Impella support initiated prior to surgery. To date, a total of 12 VSR repair procedures have been performed under this strategy, with all patients receiving preoperative Impella CP support. All 12 patients represent a consecutive series of surgical VSR repairs performed at our institution under an Impella bridging strategy. Only one patient was managed without Impella support and received IABP support instead, because Impella placement was not feasible due to stenosis of the femoroiliac access route. This patient was excluded from the present study. Inhaled nitric oxide was not administered routinely but was initiated selectively in patients with elevated mean pulmonary artery pressure and/or impaired oxygenation requiring escalation of respiratory support. During iNO therapy, pulmonary artery pressures, systemic hemodynamics, and respiratory status were closely monitored, and iNO was promptly down-titrated or discontinued if pulmonary congestion worsened.

This study was approved by the institutional review board (Approval No. 2024-048), and the requirement for informed consent was waived because of the retrospective design. The present study was conducted in accordance with the ethical standards of the responsible committee and with the Helsinki Declaration of 1964 and all subsequent revisions.

### Device

The Impella CP is a percutaneous, temporary, microaxial flow pump designed to provide left ventricular assistance. The Impella is inserted in a retrograde manner across the aortic valve into the left ventricle, where it ejects blood from the left ventricle into the ascending aorta, thereby reducing left ventricular afterload and improving systemic perfusion. In all cases, device insertion was performed via the femoral artery using a standard percutaneous technique in collaboration with the cardiology team. Appropriate positioning was confirmed under fluoroscopic and transthoracic echocardiographic guidance. Anticoagulation therapy and device management were conducted in accordance with our standardized institutional protocol.

## Anticoagulation

At our institution, during preoperative Impella support, systemic unfractionated heparin was administered, and heparin was added to the purge solution, with anticoagulation managed to maintain an activated clotting time (ACT) > 160 s. In patients with relatively low activated partial thromboplastin time (APTT) values, the target ACT was individualized, with a goal of approximately 200 s depending on the clinical scenario.

In the immediate postoperative period, a heparin-containing purge solution was generally continued; however, in patients with a high risk of bleeding, a heparin-free sodium bicarbonate purge solution was used. Postoperative systemic heparinization was initiated approximately 24 h after surgery, once acceptable chest drain output had been confirmed, with anticoagulation adjusted based on both ACT and APTT values. Despite these strategies, thrombus formation was observed in some cases even when the purge solution concentration and ACT were maintained within the target range (150–200 s).

## Device weaning, and explantation

Impella support was continued postoperatively to facilitate ventricular unloading and, when necessary, separation from cardiopulmonary bypass. Thereafter, weaning was individualized based on the severity and etiology of postoperative cardiogenic shock, aiming to preserve hemodynamic stability and adequate end-organ perfusion. Weaning was initiated after clinical stabilization with evidence of improving end-organ perfusion.

Pump support was reduced stepwise by 1 P-level at a time, with reassessment at each step using hemodynamic variables, including cardiac output/cardiac index and pulmonary artery pressures when available, along with perfusion indices such as lactate, urine output, and liver/renal function. Transthoracic echocardiography was additionally used at the discretion of the treating team to confirm device position and to assess ventricular function.

Explantation was considered when the patient maintained sustained hemodynamic stability and preserved perfusion throughout down-titration to P2 on minimal vasoactive support, without recurrent pulmonary congestion, worsening acidosis, or escalating vasoactive requirements. If instability occurred during weaning, the support level was increased and re-attempted after further stabilization.

## Surgical procedure

All operations were performed using the extended sandwich patch technique, which is the standard procedure at our institution, as previously described [3, 4]. Left ventriculotomy was avoided. Double-patch closure in a “sandwich” configuration was performed using large interrupted horizontal mattress sutures to provide complete, leak-free coverage of the VSR. This approach was applied for both anterior and posterior defects. In anterior cases, the right ventriculotomy was made parallel to the left anterior descending artery; in posterior cases, the incision was made parallel to the posterior descending artery.

Prior to aortic cross-clamping, the absence of aortic pathology was confirmed by epi-aortic echocardiography, and the Impella pump speed was reduced to P1. Myocardial protection was achieved with combined antegrade and retrograde cold blood cardioplegia. After the initial antegrade dose, the Impella was switched to surgical mode.

**Fig. 1:**
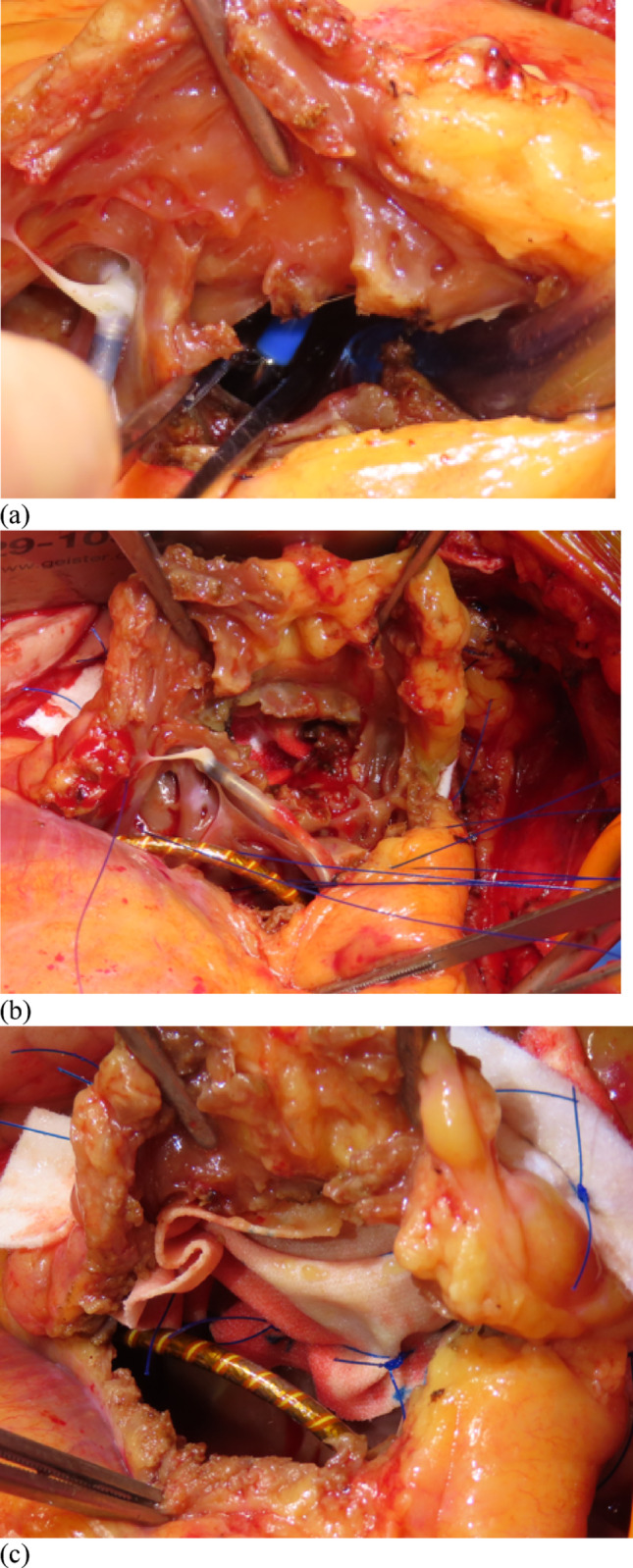
**a** The Impella device is visualized on the left ventricular side, with the inflowcannula appropriately located within the cavity. **b** Meticulous care is taken to avoid entanglement or injury to the Impella device while the left ventricular cavity is visualized and the first patch is sutured using 3−0 monofilament sutures on MH needles. **c** The lower half of the second patch is tied within the right ventricular cavity, while the upper half of the right ventricular side patch is anchored with sutures in the free wall of the left ventricle.

Dacron patches (Ethicon, Somerville, NJ, USA) were prepared to traverse the septal defect into the left ventricle. The patch size was tailored to ensure sufficient overlap with the defect margins and to fit the surrounding tissue. Necrotic myocardium at the defect rim was extensively debrided. Suturing was performed with caution to avoid entanglement with the Impella device (Fig. 1). Following closure, the left ventricle was de-aired.

Before separation from cardiopulmonary bypass (CPB), the Impella was returned to P1 mode; support was then increased to facilitate CPB weaning. The need for concomitant coronary artery bypass grafting (CABG) was determined on a case-by-case basis.

## Data collection and analysis

Clinical data, including the time from myocardial infarction onset to the diagnosis of VSR, laboratory findings, and hemodynamic parameters, were retrospectively obtained from electronic medical records. Outcome measures included 30-day mortality, major bleeding, early residual shunt flow, intestinal ischemia, and prolonged ventilation. Descriptive statistics were expressed as median values with interquartile ranges or as counts with percentages, as appropriate. Pre- and postoperative serum lactate levels, aspartate aminotransferase (AST), alanine aminotransferase (ALT), and lactate dehydrogenase (LDH) levels were compared as paired data. Normality of paired differences was assessed using the Shapiro–Wilk test. Because the paired differences were not normally distributed, the Wilcoxon signed-rank test was used for these comparisons. All statistical analyses were performed using SPSS software (IBM Corp., Armonk, NY, USA). A two-sided p value < 0.05 was considered statistically significant.

## Results

### Preoperative characteristics

The preoperative characteristics of the study cohort are summarized in Table 1. In this 12-patient cohort, the mean age was 75 ± 8 years and 50% were female. The mean body mass index and body surface area were 24.3 kg/m² and 1.61 ± 0.24 m², respectively. Comorbidities included smoking (50%), diabetes (33%), and hypertension (50%). The mean estimated glomerular filtration rate was 46 ± 23 mL/min. At presentation, 50% of patients had cardiogenic shock; preoperative support comprised IABP in 50% and ECMO in 25%, and no patient had prior cardiac surgery. Anterior-type VSR occurred in 50% of patients (defect size 18 ± 14 mm). Culprit-lesion percutaneous coronary intervention was performed in 75% of patients. The median onset-to-presentation interval was 72 h, and the mean admission-to-surgery interval was 64 ± 35 h. There were no cases requiring preoperative transfusion attributable to device-related complications.

**Table 1:** Preoperative characteristics

Variable	
Age, years	75 ± 8
Female (%)	6 (50)
Body mass index, kg/m^2^	24.3
Body surface area, m^2^	1.61 ± 0.24
Smoking history	6 (50)
Diabetes mellitus	4 (33)
Hypertension	6 (50)
Estimated glomerular filtration rate, ml/minute	46 ± 23
Cardigenic shock	6 (50)
Intra-aortic balloon pump support	6 (50)
Preoperative ECMO support	4 (25)
Previous cardiac surgery	0 (0)
Anterior VSR	6 (50)
Perforation size, mm	18 ± 14
Inhaled nitric oxide	9 (75)
Continuous hemodialysis and filtration	4 (33)
Culprit revascularization by PCI	9 (75)
Duration from AMI to hospital presentation, hours	72
Interval between admission and surgery, hours	64 ± 35
Preoperative RBC/FFP/PC transfusion, units	4 / 0 / 0

Categorical data are presented as n (%). Continuous data are presented as mean ± standard deviation

ECMO, extracorporeal membrane oxygenation; VSR, ventricular septal rupture; PCI, percutaneous coronary intervention; AMI, acute myocardial infarction. RBC, red blood cells; FFP, fresh frozen plasma; PC, platelet concentrates. Transfusion volume is expressed in units as supplied by the Japanese Red Cross and are reported as median (interquartile range)

## Operative details

The operative details are summarized in Table 2. Most patients underwent VSR repair using the double sandwich patch technique (92%; *n* = 11). Concomitant procedures comprised CABG in two patients (17%) and the Dor procedure in one patient (8%). No patients required aortic valve replacement. The mean operative time was 267 ± 66 min. The mean CPB time and aortic cross-clamp time were 139 ± 33 min and 80 ± 16 min, respectively. The mean intraoperative blood loss was 1822 ± 1046 mL.

**Table 2:** Operative details

Variable	
Double sandwich patch technique Concomitant procedures	11 (92)
Aortic valve replacement	0 (0)
Coronary bypass graft	2 (17)
Dor procedure	1 (8)
Operation time, minutes	267 ± 66
Cardiopulmonary bypass time, minutes	139 ± 33
Circulatory arrest time, minutes	80 ± 16
Intraoperative RBC/FFP/PC transfusion, units	9 / 7 / 10
Intraoperative blood loss, mL	1822 ± 1046

Categorical data are presented as n (%). Continuous data are presented as mean ± standard deviation

RBC: red blood cells; FFP: fresh frozen plasma; PC: platelet concentrates. Transfusion volume is expressed in units as supplied by the Japanese Red Cross and are reported as median (interquartile range)

### Early outcomes

The early outcomes are summarized in Table 3. Among the 12 patients who underwent Impella-bridged VSR repair, the 30-day mortality was 8%. Early residual shunt occurred in 33% of patients (reoperation in 1). The complications were pneumonia in 17% of patients, mediastinitis in 25%, bleeding reintervention in 25%, intestinal ischemia in 25%, and device-related complications in 8%. No patient underwent pacemaker implantation, but all patients required prolonged ventilation. We defined prolonged ventilation as postoperative mechanical ventilation lasting more than 48 h. Perioperative transfusion requirements were evaluated across three predefined time windows: preoperative, defined as the interval from Impella insertion to immediately before the start of surgery; intraoperative, defined as the period from skin incision to completion of surgery; and postoperative, defined as the interval from the end of surgery through postoperative day 7 (POD 0–7).

**Table 3:** Early outcomes

Variable	*n* (%)
30-day mortality	1 (8)
Days from surgery to device removal	4
Early residual shunt flow	4 (33)
Pneumonia	2 (17)
Intestinal ischemia	3 (25)
Device-related event	1 (8)
Prolonged ventilation (> 48 h)	12 (100)
Bleeding necessitating reoperation	3 (25)
Pacemaker implantation	0 (0)
Mediastinitis	3 (25)
Postoperative RBC/FFP/PC transfusion, units	6.5 / 0 / 10

We defined prolonged ventilation as postoperative mechanical ventilation lasting more than 48 h. RBC, red blood cells; FFP, fresh frozen plasma; PC, platelet concentrates. Transfusion volume is expressed in units as supplied by the Japanese Red Cross and are reported as median (interquartile range)

^a^The Impella device is visualized on the left ventricular side, with the inflow cannula appropriately located within the cavity

Only one patient in our study died within 30 days after surgery following the onset of low-output syndrome after Impella removal. The patient developed ventricular septal rupture after myocardial infarction, with the right coronary artery identified as the culprit lesion, and was referred to our institution approximately 24 h after symptom onset. Upon admission, Impella CP support was initiated immediately. After approximately 4 days of Impella-assisted stabilization, surgical VSR closure was performed. On postoperative day 3, the patient was receiving low-dose dobutamine (approximately 3 µg/kg/min) and maintained a mean arterial pressure of approximately 70 mmHg with stable hemodynamics, and the Impella was removed. Subsequently, blood pressure gradually declined with a concomitant increase in serum lactate levels. An intra-aortic balloon pump was inserted, and contrast-enhanced computed tomography was performed; however, no structural pathology sufficient to explain the shock state was identified. Transthoracic echocardiography demonstrated no recurrence of the VSR shunt. Left ventricular ejection fraction was approximately 40%, and despite escalation of catecholamine support and fluid resuscitation, metabolic acidosis did not improve, and the patient died. The underlying cause of deterioration remained unclear.

### Changes in laboratory markers during impella support

**Fig. 2:**
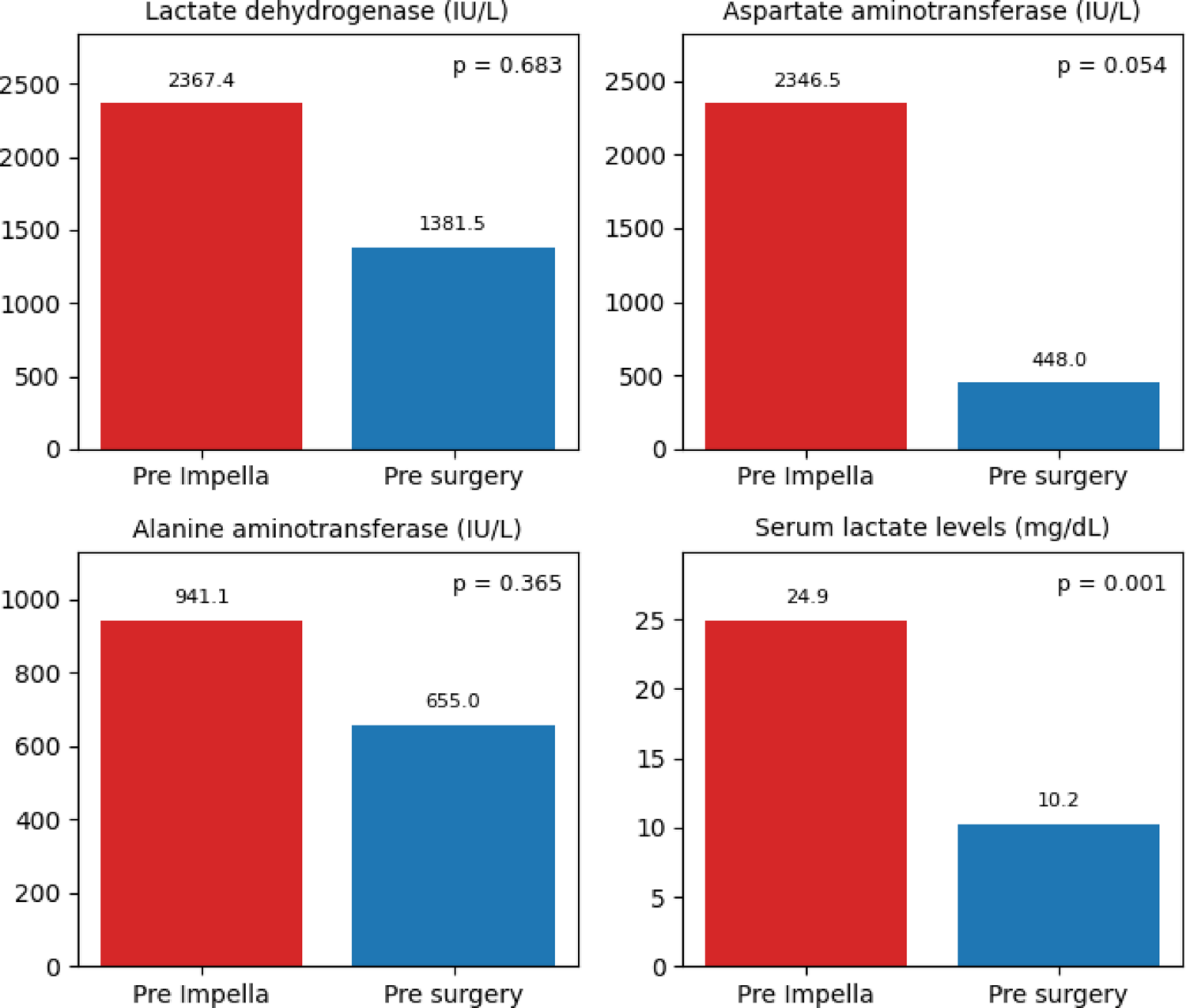
Changes in the mean serum concentrations of lactate dehydrogenase, aspartate aminotransferase, alanine aminotransferase, and lactate during Impella support

Biochemical markers substantially improved during Impella support (Fig. 2). *Serum lactate levels significantly decreased from before Impella insertion to prior to surgery (24.9 vs. 10.2 mg/dL*, *p* = 0.001). Aspartate aminotransferase (AST) levels showed a trend toward significance, decreasing from 2346 to 444 IU/L (*p* = 0.054). In contrast,* no statistically significant differences were observed in alanine aminotransferase (ALT) levels (941 vs. 656 IU/L*, *p* = 0.365) or lactate dehydrogenase (LDH) levels (2307 vs. 1381 IU/L, *p* = 0.683). Most patients demonstrated decreased AST and ALT levels by the time of surgery. However, one patient with preoperative intestinal ischemia showed persistently marked transaminase elevation: AST decreased only modestly from 2,788 IU/L preoperatively to 2,063 IU/L at surgery, whereas ALT increased from 2,098 IU/L to 3,353 IU/L. This outlier case contributed to the overall higher perioperative transaminase values observed in the cohort.

## Discussion

VSR is a critical condition characterized by abrupt left-to-right shunting, which often leads to low-output syndrome. The Impella device aspirates blood directly from the left ventricle and ejects it into the ascending aorta, and thus offers a potential pathophysiologic advantage in patients with VSR by directly unloading the left ventricle and improving systemic perfusion. However, reports on the use of Impella support for VSR remain scarce. In the present study, all 12 patients proceeded to surgical repair regardless of their preoperative condition, and the 30-day mortality was limited to 8% (one patient), representing favorable early outcomes compared with prior studies. Jalli et al. reported a mean interval of 10.5 days between Impella implantation and VSR repair, with an overall 30-day mortality of 46.2%, including conservatively managed patients [5]. Similarly, the CAUTION trial reported a short-term mortality of 58% [6]. Saito et al. described favorable outcomes using the ECPELLA in patients with mechanical complications of cardiogenic shock, including three patients with VSR cases; all three patients underwent delayed surgery after an average of 5 days and survived the early postoperative period [7]. In the present study, four patients (including one with preoperative cardiopulmonary arrest) received ECPELLA support; all four survived to 30 days, and three remain alive to date.

The optimal timing for surgical intervention in patients with VSR remains uncertain. The CAUTION trial reported that the outcomes improve if surgery can be delayed for more than 1 week [6]. Therefore, the European guidelines recommend a shift from emergency surgery to delayed elective VSR repair [8]. Prior to the introduction of the Impella device at our institution, emergency surgery was routinely performed for VSR; however, we now typically allow for several days of stabilization before proceeding with surgery. Specifically, at our institution, the timing of surgery under Impella CP bridging is determined by balancing (i) stabilization of end-organ perfusion and metabolic status and (ii) avoidance of clinical deterioration associated with prolonged femoral Impella support, including access-site complications, infection, bleeding, hemolysis, and lower-limb ischemia, in addition to the risk of abrupt hemodynamic collapse in patients with unstable VSR. Accordingly, rather than targeting a fixed waiting period of 1–2 weeks solely to improve tissue integrity, we proceed to definitive repair once systemic stabilization has been achieved, as assessed by serial lactate trends, decreasing vasopressor requirements, adequate urine output, and improvement in congestion. Furthermore, because Impella CP is incorporated into our postoperative management strategy, we generally plan the treatment course such that the total duration of Impella support (pre- and postoperative combined) is approximately one week. Early surgical intervention was considered a potential contributor to residual shunt formation in our series. In the acute phase, the demarcation between viable myocardium and infarcted tissue is often indistinct, which may compromise secure closure. Among the 12 patients, only one required reoperation. In that case, the full-thickness sandwich closure remained intact without disruption; however, a separate defect developed adjacent to the patch. A plausible mechanism is stress concentration in the surrounding fragile myocardium due to relative fixation of the patched segment, resulting in a secondary rupture near the repair site. To mitigate this mechanism, in patients in whom repair can be safely deferred, a longer preoperative stabilization period should be considered, including upgrading to Impella 5.5 to provide more durable mechanical circulatory support while awaiting tissue maturation and improved tissue integrity. Moros et al. reported the use of the Impella 5.5 in nine patients with VSR and the Impella CP in one patient with VSR [9]. Although three patients did not survive to surgery, those who underwent surgical VSR repair had a median mechanical circulatory support duration of 11.5 days and favorable postoperative outcomes [9]. While careful patient selection and consideration of the optimal waiting period are essential, Impella support may represent a valuable adjunct to safely bridge patients to surgery.

The present study may have achieved favorable early outcomes because it is easier to wean patients from CPB under Impella support. Patients with profound cardiogenic shock can be particularly challenging to wean from CPB, and recourse to IABP or ECMO was often unavoidable before the introduction of the Impella. The RECOVER I trial of patients who developed cardiogenic shock or low cardiac output syndrome following CPB separation demonstrated that early Impella implantation yielded prompt hemodynamic stabilization, with the pulmonary artery diastolic pressure decreasing from 28.0 ± 3.9 mmHg to 19.8 ± 3.2 mmHg [10]. This intervention facilitated recovery from post-CPB low-output states and was associated with improved early outcomes [10]. In the context of severe mechanical complications of AMI, including VSR, preoperative Impella insertion may thus serve as a valuable adjunct to achieve successful CPB weaning.

Another factor that may have contributed to the favorable early outcomes in our study was the use of the Impella as a postoperative mechanical circulatory support device for several days. Indeed, the 30-day mortality rate was low at 8%. However, it remains unclear which subset of patients derives the greatest benefit from Impella support. Morimura et al. reported that the combination of IABP and venoarterial ECMO for VSR complicated by cardiogenic shock resulted in an operative mortality of 12.5% [11]. Although comprehensive data on Impella management in VSR are scarce, a pooled analysis of 20 publications including 68 patients in whom Impella was implanted after the diagnosis of post-infarction ventricular septal defect reported a total in-hospital mortality of 47%, with 29 Impella-related complications documented [12]. Furthermore, in our study, there were reductions in the serum concentrations of aspartate aminotransferase, alanine aminotransferase, lactate dehydrogenase, and lactate during the waiting period from Impella implantation to surgery, which may also have contributed to the favorable early outcomes. However, whether postoperative Impella support should be used in all patients and the optimal duration of support remain uncertain and warrant further investigation.

At our institution, inhaled nitric oxide (iNO) is administered perioperatively when clinically indicated. Although initially established in neonatal and pediatric cardiac care, its use has expanded to adult cardiac surgery with growing evidence of potential benefit [13, 14]. In the present study, nine patients (75%) received preoperative iNO. Importantly, iNO was not used routinely for VSR but was reserved for selected patients with suspected pulmonary hypertension and/or right ventricular (RV) pressure overload requiring adjunctive pulmonary-selective vasodilation. In such situations, iNO can reduce pulmonary vascular resistance and pulmonary artery pressure without systemic hypotension, thereby decreasing RV afterload and potentially improving right ventricular–pulmonary arterial coupling (RV–PA coupling) and oxygenation.

Nevertheless, because post-infarction VSR is typically dominated by pulmonary overcirculation and left-sided dysfunction, indiscriminate pulmonary vasodilation may increase pulmonary venous return and potentially worsen congestion. When the left heart cannot accommodate the increased return, left atrial pressure or pulmonary capillary wedge pressure may rise, which can aggravate pulmonary congestion. Therefore, iNO was administered cautiously under close hemodynamic and respiratory monitoring while left-sided loading conditions were concurrently controlled by LV unloading with Impella and strict volume management, and it was down-titrated or discontinued if signs of worsening congestion emerged.

Beyond hemodynamics, NO may modulate inflammatory responses, including attenuation of postoperative IL-6 elevation and ischemia–reperfusion injury [15–17]. However, the clinical impact of iNO in VSR remains uncertain; further studies are warranted to better define appropriate indications and to clarify its efficacy and safety in this setting.

In our department, the Impella CP was used in all patients with VSR, primarily due to its ease of use. The Impella CP can be inserted percutaneously at the time of admission, whereas the Impella 5.5 generally requires surgical implantation via a prosthetic graft to the subclavian artery. During the waiting period, bleeding from the axillary access site can present a clinical challenge. Nevertheless, there have been reports of preoperative Impella 5.5 implantation allowing for prolonged stabilization prior to surgery. Shehzad et al. described a case in which the Impella CP was exchanged for the Impella 5.5 during the perioperative period; the patient was extubated preoperatively, underwent rehabilitation, and subsequently underwent VSR repair at 28 days after the onset [18]. The future management of VSR will require a tailored treatment approach that considers the advantages and disadvantages of each device.

Despite its advantages, the Impella also has associated drawbacks. There have been reports of right-to-left shunting in patients with VSR managed with the ECPELLA after the development of concomitant right ventricular failure [19]. Although no such cases were encountered in our study, vigilance is warranted in patients with suspected oxygenation impairment. In such scenarios, meticulous adjustment of the flow between the Impella and ECMO is essential. Access site complications can also present a significant challenge. In one case, infection at the Impella insertion site led to massive hemorrhage from the common femoral artery. Although we did not experience thromboembolic events attributable to a thrombus adherent to the Impella, we routinely clamped the distal common femoral artery during device removal, performed a small-volume flush, and ensured that debris did not embolize distally to the lower extremities. *Nevertheless*,* in our cohort*,* thrombus formation along the shaft of the device was frequently observed at explantation.* Sugiura et al. reported that 10 of 67 patients who underwent Impella 5.0/5.5 implantation required device reimplantation, with pump thrombosis accounting for half of these cases [20]. The lack of well-defined guidelines for anticoagulation management during Impella support may contribute to the high incidence of thrombotic complications in the literature [21].

Although the relationship between Impella use and mesenteric ischemia is not well established, 3 of 12 patients in our study developed postoperative mesenteric ischemia. Previous research has demonstrated that, among 10 patients undergoing CABG, allocation to a non-pulsatile CPB group was associated with greater reductions in gastric mucosal perfusion and more pronounced decreases in gastric mucosal pH compared with the pulsatile group [22]. An excessive Impella flow may reduce the native left ventricular ejection and narrow pulse pressure. A marked reduction in pulse pressure may approximate a non-pulsatile state, potentially leading to similar pathophysiologic consequences. Based on these observations, we have adopted perioperative management strategies aimed at maintaining pulse pressure whenever feasible.

### Limitations

The primary limitation of this study is its small sample size, which precludes definitive conclusions. However, to our knowledge, this represents the largest series to date describing perioperative Impella use in patients undergoing surgical repair of VSR. Additional limitations include the relatively short follow-up period, the single-center retrospective design without an institutional control group for direct comparison, and potential heterogeneity in surgical techniques and perioperative management across cases. There was also considerable heterogeneity in the severity of VSR, with the potential for substantial selection bias.

## Conclusions

The 30-day mortality rate of patients with VSR managed under Impella support in our study was lower than that reported in previous studies. Impella support facilitated preoperative optimization of the systemic status and provided a safe bridge to definitive VSR closure. However, the optimal timing of surgery, strategies to improve mid- and long-term outcomes, and direct comparisons with alternative mechanical circulatory support devices warrant further investigation.

## Data Availability

Data are available from the corresponding author on reasonable request.
